# The perceived effects of generational diversity on supervision of new professional nurses in public hospitals

**DOI:** 10.4102/hsag.v28i0.2227

**Published:** 2023-07-11

**Authors:** Kholofelo L. Matlhaba

**Affiliations:** 1 Department of Health Studies, College of Human Sciences, University of South Africa, Pretoria, South Africa

**Keywords:** diversity, generational diversity, new professional nurse, public hospital, supervision

## Abstract

**Background:**

The current global nursing workforce is a combination of personnel from three different generation cohorts, which are the Baby Boomers, Generation X and Generation Y (Millennials). These generational cohorts work side by side to provide quality nursing care to the patients on a daily basis.

**Aim:**

This article aims to report the effects of generational diversity on the supervision of new professional nurses in selected public hospitals in North West Province.

**Setting:**

This study was conducted at seven public hospitals situated at three out of the four districts of North West Province, South Africa. These public hospitals classifications consist of six districts and one provincial hospitals.

**Methods:**

The study followed an exploratory, descriptive and contextual qualitative research design underpinning the constructivist paradigm was followed to pave a way for this study. Operational managers were purposively sampled and data were collected using the focus group discussion as well as individual interviews. Data analysis followed the guideline of the thematic analysis.

**Results:**

This article reports on three themes that emerged from data analysis, namely (1) generational differences, (2) insubordination and (3) impact on supervision.

**Conclusion:**

Understanding the generational diversity and its impact on the supervision of new professional nurses might assist in improving the leadership styles for operational managers and will promote collegiality among colleagues and positively influence the provision of quality care for patients.

**Contribution:**

These results provide a framework for future research and provide the basis for understanding the impacts generational diversity has on supervision of new professional nurses.

## Introduction

The global nursing workforce is a combination of personnel from three different generation cohorts, which include the Baby Boomers, Generation X and Generation Y (Millennials) (Campbell & Patrician 2020; Stevanin et al. 2020). These generational cohorts work side by side to provide quality nursing care to the patients on a daily basis (Pawlak, Serafin & Czarkowska-Pączek 2022). Each generation of nurses is characterised by their experiences and expectations when it comes to responsibilities in the workplace. The Baby Boomers generation (1945–1964) grew up in a healthy post-war economy, where jobs were widely available. Baby Boomers are considered the most influential group with a direct influence on the development of Generation X. Generation X (1965–1980) is characterised by resourcefulness and independence. They usually look for work–life balance. Members of Generation Y were born between 1981 and 2001 and this generation experienced a significant influence of technology and they are more likely to be educated as compared to previous generations (Lappeman, Egan & Coppin 2020).

## Background

The Generation Y cohort comprises the young generation among the nursing workforce in the healthcare facilities in South Africa and mostly forms part of the new professional nurses entering the workplace. According to the statistics from the South African Nursing Council (SANC 2021a), 27% Registered Nurses and Midwives are the members of the Generation Y cohort with the Baby Boomers and the Generation X divided among the remaining 73%. For the purpose of this study, the three generations will be announced as the new and the old generation. In South Africa, someone with extensive years of experience occupies the nursing management position in the public healthcare facilities after registration with the SANC. It is safe to say that the majority of those in the managerial positions fall under the age of 45–65, which makes the 73% of older generation of nurses according to the SANC statistics (SANC 2021a). It is safe to say from the above-mentioned statistics; the evidence of generational diversity will be clearly perceived. Generational diversity, including workforce differences in attitudes, beliefs, work habits and expectations has proven to be challenging for nursing leaders. An acceptance of generational diversity in the workplace allows a richer scope for practice as the experiences and knowledge of each generation in the nursing environment creates an environment of acceptance and harmony facilitating the retention of nurses. Hisel (2020) suggests that nurse leaders should take full advantage of the experience of the retiring generations to mentor and transfer critical knowledge to the Gen X and millennial nurses.

## Problem statement

During the transition period, a new professional nurse relies on the support and mentoring of an experienced nurse to fully develop the skills and abilities to function as an independent nurse practitioner. However, the noticeable impact caused by the age differences among the nursing profession cannot be overlooked. The researcher observed the challenges brought in by the generational gaps and diversity in the nursing profession during data collection for her research work in the past 5 years. From the current research project, ‘Enhancing clinical competence of young professional nurses through collaboration in the North West Province, South Africa’, aiming to develop a programme to enhance clinical competence of new professional nurses led to the researchers’ decision to look at the generational diversity, its impact as well as measures of dealing with it in the workplace. From the current research work, participants raised concerns about the generational gaps and diversity in the nursing profession, which some perceived to be an obstacle in having competent and independent nursing practitioners. This prompted the researcher to report perceived generational gaps and diversity and its effects on supervision and to make recommendations. Therefore, this article aims to report the effects of generational diversity on supervision of new professional nurses in selected public hospitals in North West Province.

## Definition of concepts

### Generational diversity

According to Stiehr and Vandermause (2017), generational diversity is characterised by the collective experiences of members of a group within a designated span of birth years (generation) that shape value sets and attitudes among members of the generation. In this study, generational diversity includes the veteran and educational status among the operational managers and the new professional nurses.

### New generation

Campbell and Patrician (2020) define a generation as a group of people born within a certain year range. In this study, the new generation of professional nurses are those nurses who are employed at the selected public hospitals under the supervision of the operational managers who are the participants in this study.

### New professional nurse

As defined by the SANC (*South Africa 2005: s 58*[***1***] [*q*]), a professional nurse is a person who:

[*H*]as met the prescribed educational requirements for registration as a professional nurse in the Regulations relating to the Approval of and the Minimum Requirements for the Education and Training of a Learner Leading to Registration in the Categories Professional Nurse and Midwife, published in Government Notice No.R. 174 of 8 March 2013. (p. 6)

In this study, a new professional nurse refers to a new nurse who has completed community service and is currently employed at selected public hospitals in the North West Province.

### Supervision

The SANC defined supervision as a professional development activity where the less experienced nurses benefit from the knowledge and experience of their supervisor which helps to address any gaps in knowledge or skill set (SANC 2021b). According to SANC, supervision for nurses can either be direct or indirect depending on the supervisee’s experience. Hence, in this study, supervision refers to the indirect professional support and guidance expected from the unit managers towards the new professional nurses working in different clinical areas of the selected public hospitals.

### Supervisor

A supervisor is a nurse manager who is responsible for ensuring the provision of clinical care in their clinical areas and has immense responsibility to ensure that organisational expectations are implemented to achieve patient and staff outcomes (Mudd et al. 2022; Penconek et al. 2021). In this study, the term supervisor refers to operational managers who are responsible providing direct and indirect supervision to new professional nurses in their units.

### Purpose of the article

This study aimed to report the effects of generational diversity on supervision of new professional nurses in selected public hospitals in North West Province.

Following on this introduction, the author then presents the methods that were used to collect and analyse the data, this is followed by a presentation of the results, followed by a discussion of the results and how they link to the literature and finally the author shall present a conclusion drawn from the results and areas for further research.

## Research methods and design

### Study design

The study followed an exploratory, descriptive and contextual qualitative research design underpinning the constructivist paradigm was followed to pave a way for this study. According to Creswell and Creswell (2017), constructivism paradigm aimed to understand the experiences of participants with the intention to discover their perceptions. The choice for this approach was grounded on the perspective of gathering a thick and rich understanding of how operational managers view the generational diversity impact on supervision of new professional nurses in the public hospitals units where they are tasked with the managerial responsibility.

### Research setting

This study was conducted at seven public hospitals situated at three out of the four districts of North West Province, South Africa. These public hospitals classification consists of six district and one provincial hospital.

### Study population and sampling strategy

Population is the set of all members of a defined group (Gray, Grove & Sutherland 2016). The sample refers to the subset of the accessible population that the researchers selected to participate in this study (Gray et al. 2016). A purposive sampling was used to purposively select the participants and to avoid interrupting healthcare service delivery; interviews took place according to participants’ availability. Hence, participants who were readily available in the units were assembled together for the focus group discussions (FGDs) or individual interviews. Forty-six operational managers (5 males and 41 females) participated in this research. Inclusion criterion for the study was professional nurses in the position of operational managers either by appointment or acting by the time of data collection in different fields of speciality of the selected public hospitals. Other professional nurses who are not occupying the position of operational managers were excluded from participating in this study.

### Data collection

Data were collected between September and November 2021 during the South African National Lockdown Level 1. COVID-19 guidelines and protocols were followed at all times during the data-collection period. Appointments were made with hospitals’ nursing service managers who then gave permission to access the units. Individual unit managers were then approached by the researchers and explained the purpose of the study. Those who agreed to participate were then interviewed. In some instances where a group of six participants or more were willing to participate, FGDs were used to gather much information at the same time as well as to save time. In some hospitals where it was not possible to get more participants, individual interviews were conducted. The FGDs were conducted in the hospitals’ boardrooms and unit manager’s offices. The two experienced qualitative researchers collected data and they had no personal relationships with the participants. With both FGDs and individual interviews, participants and researchers were seated at a minimum social distance of 1-m apart, wearing masks covering both the mouth and the nose and were sanitised. In this study, data were collected by using four FGDs as well as seven individual interviews subject to the number of participants available in the study settings. The focus groups consisted of participants of between 6 and 13 operational managers, with interviews that lasted for a minimum of 50 min and beyond an hour, whereas individual interviews lasted between 30 and 45 min. Interviews were conducted in English. In instances where the vernacular language was used, translation was performed during transcription. Field notes were also captured for direct observations on behaviours of participants during interviews and complemented the audio records. Data saturation was reached with the fourth focus group as a confirmation of no new information emerged from the participants (Creswell & Creswell 2017).

### Data analysis

This study followed the six-phase guide of the thematic analysis proposed by Braun and Clarke (2006). Thematic analysis is an approach to identify, analyse and report the patterns from datasets. The audio recordings were transcribed verbatim, which was later analysed manually by the two researchers. Data analysis followed the following steps: firstly, familiarisation of data by deeply scrutinising the data through re-reading of the transcripts, notes and highlighting similar ideas. Secondly, initial codes were manually generated and the list on the interesting ideas were noticed. Thirdly, themes were searched after coding the initial themes and overarching subthemes. Fourthly, themes were reviewed and refined. Fifthly, themes were defined and named and accompanied by the extracts from the transcripts. Lastly, a report was produced for discussion.

### Measure of trustworthiness

This study was guided by the qualitative principles of trustworthiness, credibility, dependability, confirmability and transferability, authenticity (Lincoln & Guba 1985). The researchers collected data and analysed it themselves to ensure credibility (Polit & Beck 2021). Finally, the researchers had a round table session discussing data and came to an agreement on the themes and subthemes, thus ensuring confirmability of the study. To ensure transferability and authenticity in this study, research methods including the study settings, sampling and data-collection techniques were clearly discussed. Furthermore, direct quotations were used to provide sufficient description of data for other researchers to establish whether the results can be transferred to their context (Polit & Beck 2021).

### Ethical considerations

University of South Africa (UNISA) College of Human Sciences Research Ethics Committee granted the ethical clearance certificate (Ref: 90388526_CREC_CHS_2021). Furthermore, permission to conduct this study was sought from all the stakeholders, namely the Department of Health North West Province, District management and Chief Executive Officers of the selected hospitals in the province. All communications regarding the study were shared with all relevant stakeholders through emails and followed by the face presentation meetings. Prior to the commencement of the interviews, signed consent forms were obtained from the participants. Consent forms were signed without coercion with a detailed report as to why the study is important for the health facilities in the North West Province. Issues of anonymity and confidentiality were reinforced as this study was purely voluntary.

## Results

### Description of study participants

The participants were operational managers at seven public hospitals in the three districts of the North West Province. The participants’ age ranges between 40 and 65. The description of participants for this study is presented in Table 1.

**TABLE 1:** Hospitals number and classification, method of interview conducted, number and gender of participants.

Hospitals number and classification	Interview method	Number of participants	Gender of participants	
			Females	Males
District Hospital 1	Individual interviews	5	4 females	1 male
District Hospital 2	Focus group discussions	13	10 females	3 males
District Hospital 3	Focus group discussions	9	9 females	0 males
District Hospital 4	Individual interviews	3	3 females	0 males
District Hospital 5	Focus group discussions	7	6 females	1 male
Provincial Hospital 6	Individual interviews	2	2 females	0 males
District Hospital 7	Focus group discussions	7	7 females	0 males

**Total number of participants**		**46**	**41 females**	**5 males**

### Presentation of themes and subthemes

Three main themes and six subthemes emerged from this study. The three themes are as follows: Enthusiastic behaviour, destructive behaviour and impact on supervision. These themes, subthemes and codes are presented in Table 2 and are described and discussed in the subsections that follow.

**TABLE 2:** Themes, subthemes and codes derived from focus group discussions as well as individual interviews.

Themes	Subthemes	Codes
Generational differences	Willingness to share new information	They come with new information and willingness to share with colleagues
	Knowledgeable about technology or computers	They assist with computer duties
Insubordination	Displays rebellious behaviour and defies the rules	They display rebellious behaviour and defy the rules and challenge the status quo
	Excessive and inappropriate use of mobile phones	They are forever on their mobile phones
Impact on supervision	A lack of trust between the supervisor and the supervisee	They cannot be trusted and they don’t trust supervisors
	Trigger intergenerational conflicts	They have judgemental attitudes towards the older nurses, which leads to disharmony among colleagues

### Theme 1: Generational differences

Participants in this study acknowledged the generational diversity and what the new professional nurses could offer in terms of contributing to patient care as they are reported to have the teachable skills. Hence, their willingness to teach and share what they know with the colleagues in the units.

Below are the subthemes and supporting quotes from participants.

#### Subtheme 1.1: Willingness to share new information

‘There is no one who know better than anyone does because they are newly qualified, they can come with new things, they can come and teach us, and we will give them whatever we have known. There is no one who is perfect when coming to nursing. Sometimes they come with new ideas. Then you take it over to your seniors and come together with the solution and say how about […] I have this, I learnt this, how about you implement this?’ (Hospital 4; individual interview 1, female participant)

Another participant had this to say:

‘So, they come with something that you were not expecting or you didn’t know but they come with something that it can come with a positive impact to the system, to the unit, to the patient. So, they impact into the units and, honestly, it’s positive, it’s different.’ (Hospital 1; individual interview 1, female participant)

#### Subtheme 1.2: Knowledgeable about technology or computers

Interestingly, contrary to the majority of studies on the use of mobile phones that have been regarded as a negative thing, some participants in this study felt the importance of the new generation of professional nurses when it comes to their skills in the use of technology and appreciated their willingness to assist. Some participants said:

‘And they are very good in technology. They are very good. You can see. Most of the things, we are not learned about. We are not using the computers; we are not using the phone. So, really, they are […] I can give them more than a hundred percent. Maybe the as OPM (Operational manager) you know nothing about this. We were used to write. Now everything is moved. You just draft then let them do the work. Things are easy.’ (Hospital 4; individual interview 1, female participant)

Another participant said:

‘Yes. They have the advantage of coming from the institution with computer literacy of which for us it was not a compulsory part. So, for them, you also gain someone who has IT knowledge, you also gain someone who say I can update this, I can do this. So, you were thinking I have to go and ask someone to type for me that. They assist. They will tell you I have a stick, I will move it from this stick to the other stick. They will give you knowledge; this is how things are done. They come with […] especially when it comes to technology, and they have that spirit. They are different.’ (Hospital 1; individual interview 2, female participant)

### Theme 2: Insubordination

Some participants in this study raised concerns regarding the behaviour of the new professional nurses who were associated with the young generation as the youngest cohort among the nursing workforce in the public hospitals. These behaviours were regarded as defiant towards supervision and if not attended to might have a negative implication on patient care delivery as well as a hostile environment, which is not conducive to the professional development of the new professional nurses as well as the well–being of the operational managers being the supervisors.

Subthemes for the above-mentioned theme are presented here along with the supporting quotes from participants.

#### Subtheme 2.1: Rebellious behaviour and defying the rules

One participant had this to say:

‘My experience with these current ones, I think they are more of being rebellious unlike us old generation. They like questioning. Almost each and every delegation you will have to watch them. They will just not perform it. They will scrutinise and take it to task, somehow defying rules in what they are doing […]. They want to be uncooperative.’ (Hospital 1; individual interview 4, male participant)

Another participant said:

‘Even though they show interest, but I’ve noticed that the main problem is, these young professional nurses, they want to change the era of nursing. They disobey the rules.’ (Hospital 5; FGD 3, participant 6, female)

#### Subtheme 2.2: Excessive and inappropriate use of mobile phones

In contrast to subtheme 1.2, some participants complained about the new generation of professional nurses’ excessive and incorrect use of technology, especially mobile phones. Some participants said:

‘You still find few, those who will be active on their gadgets. They want to give care while they are using gadgets. These cell phones of theirs […] While they are working, while they are going and see patients, pushing a trolley, having a phone and when you ask her are you supposed to be doing that while you are doing what you are doing, they’ll look at you as if you are being funny.’ (Hospital 1; individual interview 4, male participant)

‘They’re always on their phones. You will try to talk to them. No, there is no […] you will have your phone at lunch/teatime, that is when you are supposed to be always on your phone. Now you are supposed to be working, you are supposed to be attending to your patient, concentrating, focussing. They will say yes but when you just turn, they will start again. That’s always the problem.’ (Hospital 5; FGD 3, participant 7, female).

### Theme 3: Impact on supervision

Supervision plays an important role in the professional development of new professional nurses as well as enhancing provision of patient care in the unit. Operational managers as supervisors has a duty to ensure that new professional nurses are sufficiently supported and guided at all times. The following subthemes depict the impact of generational diversity on supervision of new professional nurses in some public hospitals as perceived by operational managers with support from the direct quotations.

#### Subtheme 3.1: A lack of trust between the supervisor and the supervisee

Some participants reported that the new professional nurses do not give them any reason to be trusted as they sometimes act irresponsibly and absent themselves from duty without valid reason.

Some participants had this to say:

‘With absenteeism, it is a big problem. They are always absent. They get sick when they have to work. It is a trend. I don’t trust them sometimes, they are not trustworthy employees. But I reprimand them.’ (Hospital 2; FGD 1, participant 10, female)

However, other participants had a different view as she reported that at times the operational managers’ behaviours are the cause of mistrust from the new professional nurses’ side:

‘They don’t know now who to trust anymore because when they go to the other side, the operational manager tells them this, when they go another staff member, the staff is telling them the other side. Yes. So, they end up getting just so fed up.’ (Hospital 2; FGD 1, participant 10, female)

‘Sometimes they don’t trust us because is like you are trying to make them to be scared of the profession because if you approach them in a negative way they’re going to think that they have made a wrong decision by choosing nursing. So, all we have to do is to try by all means and motivate them to become our future nurses because of as the years pass by we are going to get out of the system, they are going to remain in the system. So, we need to try by all means to keep them focussed and positive and support them.’ (Hospital 4; individual interview 2, female participant)

#### Subtheme 3.2: Trigger intergenerational conflicts

Some participants reported that the new professional nurses had a tendency to trigger intergenerational conflicts, which have a negative impact on supervision as it affects their working relationships. Some of the participants’ quotations are discussed next.

**One participant said:**

‘When you talk to them, some of them they call us antique, ancestors. Whatever we are doing or whatever they are doing to us, it is not a good thing, especially when in maternity. In addition, after maybe we have that argument, tomorrow you find that she is sick, she is not on duty. She brings a sick note the next day. Because they are not happy and it is not their fault […] and it is not our fault. I wish there could be that incorporation of ancestral and the new generation.’ (Hospital 7; FGD 4, participant 1, female)

Another participant supported the given statement by saying this:

‘Even talking to them is a challenge because we must try to build the relationship with them, because sometimes they come with this concept, like sister has already mentioned that we are ancestors, and ancestral as we are we must just try to be nice and talk to them, and build the relationship.’ (Hospital 5; FGD 3, participant 4, female)

## Discussion

This article aimed to report the perceived effects of generational diversity on the supervision of new professional nurses in selected public hospitals in North West Province. The new professional nurses are bringing the new expectations and ideals about life and work into healthcare work settings. Interestingly, the groups of operational managers who participated in the study comprised both the old and new generation and this is what the two generations of operational managers viewed the new professional nurses under their supervision. Ay and Polat (2022) suggested that if nurse managers can embrace generational differences and allow each generation to benefit from each other’s experiences and knowledge, this can contribute to the individual nurse’s development and could create greater connection as well as collaboration among the nursing teams. This is the case in this study whereby operational managers reported that they could benefit from the new professional nurses experiences and expertise when it comes to their presentation skills, the use of technology and computers. From this study, it is obvious that the relationships between the new professional nurses being the new generation and the operational managers being the older generation is diverse, which affects the supervision and mentoring of new professional nurses. Pawlak et al. (2022) emphasised that nurses cooperating in multigenerational teams have the opportunity to share their professional experience, knowledge and skills regarding the use of technological innovations. However, from this study, not all the aspects of cooperation were reported.

From the results of this study, one can argue that there are indeed implications on supervision of young professional nurses’ caused by generational gaps and diversity among the nursing workforce at the selected public hospitals, whether negative or positive. The misjudgement of characters reported, reflected the manner in which new professional nurses are viewed by their supervisors being the operational managers. According to Stiehr and Vandermause (2017), generational differences can lead to a lack of understanding, compromised communication and decreased productivity. Furthermore, it was reported that generational differences can indeed trigger conflicts that affect the employees’ performance, which was evident in this study.

On the contrary, the participants of this study reported a positive impact of generational gaps and diversity in the form of appreciation and acknowledgement of the fact that the new professional nurses are technologically inclined and that is of benefit for the issues of ward administration. One of the main roles of the operational manager is to compile a monthly report or to prepare statistics on patients’ admission. Lately, the South African government has introduced the electronic health information system in public hospitals of the country (Wright, O’Mahony & Cilliers 2017). This means that all employees will be expected to be competent with the use of technology as one of the strategies for the country towards improving the quality of public healthcare services as well as improving the accessibility of these services (Katurura & Cilliers 2018). In a study conducted by Armstrong, Rispel and Penn-Kekane (2015) on whether the initiatives of nursing unit managers facilitate provision of quality patient care in South Arican hospitals, it was reported that unit managers from those hospitals where the electronic patient information system was employed felt that their workload was eased considerably. According to this study, majority of the operational managers are not competent enough with the use of technology and they rely on the young generation for assistance. It is therefore noteworthy to highlight that the participants in this study acknowledged that even though they are having some concerns on the excessive use of mobile phones while on duty, they are of great assistance when it comes to the use of computers for typing reports and statistics as well as other related activities.

According to Helaß et al. (2022), ageing nurses are exposed to age stereotyping and ageism, which are stressors and risk factors that can affect job satisfaction and teamwork among the nursing colleagues. This statement supports the results of this study as the operational managers were called names because of their ages while trying to reprimand the new nurses. This was perceived to be the major cause of conflicts and barrier to a healthy working relationship among the nursing colleagues. In addition, these results further support the results of the analysis by Pawlak et al. (2022), which reported that the generational diversity in the workplace could lead to conflicts as it was reported in this study. Participants reported that at times they are not eager to discharge their responsibilities as supervisors because of the conflicts between themselves and the new professional nurses.

On the other hand, in a review conducted by Rothwell et al. (2021), the authors reported that unhelpful and untrusting relationships led participants to distrust their supervisor’s advice. Therefore, there is a need for supervisees to feel that they can trust their supervisors (Rothwell et al. 2021). These results concur with this study as it was pointed out that there is a lack of trust among the parties. Furthermore, the results of this study concur with the results of the study conducted in Sweden by Bry and Wigert (2022). The study reported that senior nurses being the supervisors perceived that there was a generational shift in attitudes concerning the way they communicate with recently graduated nurses as they felt that new professional nurses could not tolerate being corrected and took things as personal (Bry & Wigert 2022). There is no doubt that this reported disharmony between the two parties might affect negatively the new professional nurses’ professional growth as well as patient care if not properly handled.

## Limitations

The main purpose of this research was to develop a programme to enhance clinical competence of new professional nurses in public hospitals through stakeholders’ collaboration. The impact of generational gaps and diversity could not be ignored despite the fact that it might be from the minority of participants, which means that the results cannot be generalised. Hence, the need for future research on the impact of generational diversity among the nursing workforce in public hospitals. Furthermore, the unavailability of operational managers in some hospitals including the provincial hospital, which led to shifting from FGDs to individual interviews data-collection method can be viewed as a limitation in this study. Another limitation is the fact that only the perceptions of operational managers was considered which might be single-sided. This sets a foundation for future research to explore and describe the perceptions from the new professional nurses’ sides as well as a comparison study to compare the results from both sides.

## Recommendations

The impact of generational diversity on supervision of new professional nurses discussed in this study cannot be ignored. It is therefore recommended that operational managers as supervisors need to understand and accept the generational diversity to diminish generational conflicts within their working spaces (Rollan Oliveira & Siles González 2021). Furthermore, it is recommended that operational managers look at their attitudes and behavior as supervisors, and acknowledge that at times that contributes to the behavior displayed by new professional nurses towards them. In addition, supervision helps to promote practitioners’ personal and professional development through fostering a supportive relationship and working coalition (Saab et al. 2021). Therefore, it is recommended that operational managers nurture the relationship with new operational managers as this will have a direct benefit on the development of new professional nurses as well as indirect benefits on patient care delivery.

## Conclusion

This article aimed at reporting the effects of generational diversity on the supervision of new professional nurses in selected public hospitals in North West Province, South Africa as perceived by operational managers. It was determined that the supervision of new professional nurses plays an important role in the professional development of the new professional nurses’ career. However, opportunities and challenges caused by generational differences during supervision could not be ignored. Therefore, it is paramount that the operational managers understand that each generation has its own morals and principles; therefore the generation of new professional nurses’ behaviour and practice will differ from their own. Nurmeksela et al. (2021) ascertain that it is critical for nurse managers to enhance nursing and patient outcomes as well as focus on improving nursing practices by managing and ensuring that their subordinates feel supported, motivated and protected at all times. Likewise, operational managers as participants in this study are expected to provide indirect supervision to the new professional nurses under their watch in order to enhance their clinical competence as well as patients’ outcomes.
